# Como Avaliar a Condução Elétrica Prejudicada do Coração Direito em Pacientes com Hipertensão Arterial Pulmonar?

**DOI:** 10.36660/abc.20250159

**Published:** 2025-05-28

**Authors:** Naoya Kataoka, Teruhiko Imamura

**Affiliations:** 1 Universidade de Toyama Segundo Departamento de Medicina Interna Toyama Japão Segundo Departamento de Medicina Interna, Universidade de Toyama, Toyama – Japão

**Keywords:** Eletrocardiografia, Insuficiência Cardíaca, Bloqueio de Ramo

**Ao editor**

A identificação de uma modalidade ideal para monitorar a gravidade da hipertensão arterial pulmonar (HAP) continua sendo imperativa. Os autores demonstraram que um intervalo RS prolongado no eletrocardiograma foi associado a desfechos clínicos adversos nesta coorte, sob a hipótese de que a condução elétrica do coração direito está comprometida em pacientes com HAP grave.^1^ No entanto, várias preocupações merecem consideração.

O intervalo RS foi medido nas derivações inferolaterais, especificamente V5 e V6.^1^ No entanto, as informações do ventrículo direito são convencionalmente derivadas das derivações precordiais direitas, como V1 e V2. Em casos de bloqueio do ramo direito, uma onda S proeminente nas derivações inferolaterais é uma característica. Um intervalo RS prolongado nessas derivações pode refletir uma onda R dominante nas derivações precordiais direitas. Estudos anteriores identificaram uma amplitude elevada da onda R na derivação V1 como um marcador prognóstico em pacientes com HAP.^2^ Observações comparáveis foram feitas na síndrome de Brugada, onde a disfunção ventricular direita predomina.^3^ A incorporação das características da derivação precordial direita pode aumentar a robustez dos achados dos autores.

Além disso, o comportamento longitudinal do intervalo RS, particularmente sua trajetória em resposta a reduções na pressão da artéria pulmonar durante intervenções terapêuticas, justifica uma exploração mais aprofundada.

Possíveis fatores de confusão não considerados na análise podem ter influenciado os resultados. Dada a longa duração do estudo, de 2010 a 2022,^1^ o cenário terapêutico para HAP provavelmente evoluiu, com mudanças nos regimes de medicação. Além disso, as respostas dos pacientes ao tratamento são significativamente moduladas por fatores genéticos, como mutações no gene BMPR2.^4^ Abordar essas variáveis pode proporcionar uma compreensão mais abrangente da relação entre o prolongamento do intervalo RS e os desfechos clínicos na HAP.
