# Supplementary material for: Como Avaliar a Condução Elétrica Prejudicada do Coração Direito em Pacientes com Hipertensão Arterial Pulmonar?
Source: Arq Bras Cardiol. 2025 May 28;(5):e20250159. [Article in Portuguese] doi: 10.36660/abc.20250159 (PMC12212389; doi:10.36660/abc.20250159)
Supplement: Translation

# Translation: How to Assess Impaired Right Heart Electrical Conduction in Patients with Pulmonary Arterial Hypertension?

**DOI:** 10.36660/abc.20250159i

**Authors:** Naoya Kataoka, Teruhiko Imamura

**Affiliations:** 1 Toyama Japan Segundo Departamento de Medicina Interna, Universidade de Toyama, Toyama – Japan

**Keywords:** Electrocardiography, Heart Failure, Bundle-Branch Block

**To editor**

The identification of an optimal modality to monitor the severity of pulmonary arterial hypertension (PAH) remains imperative. The authors demonstrated that a prolonged RS interval on the electrocardiogram was associated with adverse clinical outcomes in this cohort, under the hypothesis that right heart electrical conduction is compromised in patients with severe PAH.^1^Nonetheless, several concerns merit consideration.

The RS interval was measured at inferolateral leads, specifically V5 and V6.^1^However, right ventricular information is conventionally derived from right precordial leads, such as V1 and V2. In cases of right bundle branch block, a prominent S wave in the inferolateral leads is a characteristic feature. A prolonged RS interval at these leads may reflect a dominant R wave in the right precordial leads. Previous studies have identified an elevated R wave amplitude in lead V1 as a prognostic marker in PAH patients.^2^Comparable observations have been made in Brugada syndrome, where right ventricular dysfunction predominates.^3^The incorporation of the right precordial lead characteristics could enhance the robustness of the authors’ findings.

Additionally, the longitudinal behavior of the RS interval, particularly its trajectory in response to reductions in pulmonary artery pressure during therapeutic interventions, warrants further exploration.

Potential confounders not accounted for in the analysis may have influenced the results. Given the extended study duration spanning from 2010 to 2022,^1^the therapeutic landscape for PAH likely evolved, with shifts in medication regimens. Moreover, patient responses to treatment are significantly modulated by genetic factors, such as mutations in the BMPR2 gene.^4^Addressing these variables may provide a more comprehensive understanding of the relationship between RS interval prolongation and clinical outcomes in PAH.
